# Ultra-Processed Food and Chronic Kidney Disease Risk: A Systematic Review, Meta-Analysis, and Recommendations

**DOI:** 10.3390/nu17091560

**Published:** 2025-04-30

**Authors:** Kristin E. Leonberg, Manish R. Maski, Tammy M. Scott, Elena N. Naumova

**Affiliations:** 1 Friedman School of Nutrition Science and Policy, Tufts University, Boston, MA 02111, USA; 2 Beth Israel Deaconess Medical Center, Boston, MA 02215, USA

**Keywords:** ultra-processed food, NOVA classification, chronic kidney disease, dietary pattern, nutrition guidelines, preventative health, systematic review and meta-analysis

## Abstract

**Background:** Ultra-processed foods (UPFs) are formulations of ingredients that are mostly of exclusive industrial use and may contain additives like artificial colors, flavors, or stabilizers. The sale and consumption of these foods have been increasing despite their associations with increased risk for several non-communicable diseases, including chronic kidney disease (CKD). Compared to less processed and perishable foods, UPFs have longer shelf stability, are widely accessible, and are convenient. They also tend to be more affordable and lower in nutritional quality. The aim of this systematic review and meta-analysis (PROSPERO ID: CRD42023488201) was to investigate whether consumption of UPF is associated with a higher risk of CKD in adults. **Methods:** We completed a systematic search using Medline, EMBASE, CINAHL, and Cochrane Central databases to identify observational studies published since the wide acceptance of UPF classification and conducted a random-effects model to pool the risk estimates. **Results:** A total of seven studies met the inclusion criteria for the systematic review, of which four were eligible for meta-analysis. Across these studies, there were 19,645 incident CKD cases from individuals free of baseline CKD. Using a random-effects model, higher UPF intake was significantly associated with increased CKD risk (pooled log-hazard ratio = 0.17; 95% CI: 0.07–0.28; *p* < 0.001). **Conclusions:** Given the substantial evidence from this systematic review and meta-analysis indicating an association between UPF and CKD, it is recommended for public health policies to address this risk. Promoting dietary guidelines that encourage the consumption of minimally processed foods could potentially mitigate the prevalence of CKD and improve overall public health outcomes.

## 1. Introduction

The explosion of evidence linking ultra-processed foods (UPFs) with numerous health outcomes underscores a growing concern across the spectrum of disease research [1]. The convergence of dietary habits and chronic kidney disease (CKD) is a subject of increased interest due to a high number of individuals affected by CKD worldwide [2]. The role of diet, particularly dietary patterns and practices including food processing, serves as a potentially modifiable risk factor for CKD incidence and progression.

Food processing is a process of changing agricultural products into food, or changing one form of food into another, and a practice to preserve and extend shelf life that includes, but is not limited to, pasteurizing, drying, canning, etc. [3]. Modern food processing involves the commercial production of ready-to-eat or heat-and-serve foods and has expanded in part to address changes in consumer preferences and the demand for convenient, palatable, and portable choices, with marketing strategies, appealing packaging, and hyper-palatability playing key roles in driving consumer appeal and consumption [4]. The extended shelf-life of UPF, achieved through additives and preservatives, further enhances their convenience and marketability, making them more accessible to consumers [5].

In general, as the degree of food processing increases, foods tend to be higher in energy density, sugar, saturated fat, and salt, while lower in dietary fiber, protein, vitamins and minerals, and nutritional quality [6,7,8]. In 2009, UPFs were first described in the scientific literature [8,9]. The NOVA classification system was further refined to include foods that are ready to consume, such as packaged snacks, soda, instant noodles, and pre-prepared dishes [10]. The NOVA classification system has become the most widely used food classification system to investigate the association between levels of UPF intake and diet quality and the potential effect on many chronic diseases [11]. Recently, there has been a marked increase in the accessibility and intake of UPF around the world [12], parallelled by a decrease in the amount of minimally processed foods [13].

The role of diet in the progression of kidney disease has been extensively studied, from individual dietary components such as sodium, potassium, and protein intake to dietary patterns including the Mediterranean diet, the Dietary Approach to Stop Hypertension (DASH), vegetarian diet profiles, and a Western Dietary Pattern [14]. The latter is characterized by increased intake of UPF with an associated decrease in nutrient-dense foods recommended by most dietary guidelines.

Despite ongoing research on diet and kidney health, a gap in knowledge remains regarding the impact of increased UPF consumption on kidney disease, particularly in dietary patterns where a high proportion of daily calories come from UPF. This systematic review and meta-analysis seeks to provide a comprehensive summary of available evidence by systematically identifying, evaluating, and synthesizing studies investigating the consumption of UPF and its associations with the incidence and progression of CKD in adults and highlighting the challenges of assessing these relationships to inform decision-making for health professionals.

## 2. Methods

### 2.1. Protocol and Register

This systematic review was conducted according to the National Academy of Medicine’s Standards for Systematic Reviews [15], the Cochrane Handbook for Systematic Reviews, and adhered to the Preferred Reporting Items for Systematic Reviews and Meta-Analyses (PRISMA) 2020 [16] and Meta-analysis Of Observational Studies in Epidemiology (MOOSE) [17] reporting guidelines. Prior to data extraction, the review protocol was registered in the International Prospective Register of Systematic Reviews (PROSPERO), under identification number CRD42023488201.

### 2.2. Data Sources and Searches

We searched the Medline (1946 to 31 July 2023), EMBASE (1966 to 31 July 2023), CINAHL (1961 to 31 July 2023), and Cochrane Central (1991 to 31 July 2023) databases to identify literature examining the relationship between UPF intake and CKD. An updated search was conducted on 24 March 2025 to capture any additional eligible publications. Studies published after 2009 were considered for inclusion, as this marked the first time UPF classification was introduced in the scientific literature [8].

The search strategy consisted of a combination of key words and database-specific controlled vocabulary describing the concepts UPF and CKD. Search results were limited to English-language and human studies. Complete search strategies are available in Supplementary Materials Table S1. We also reviewed the reference lists of relevant review articles to avoid missing any relevant publications and identify additional studies [18]. The search strategy was intentionally broad to ensure that no relevant publications were missed, given that UPF is a relatively new construct and the scientific field examining its health impacts remains in an early stage of development.

### 2.3. Study Selection

**Figure 1:**
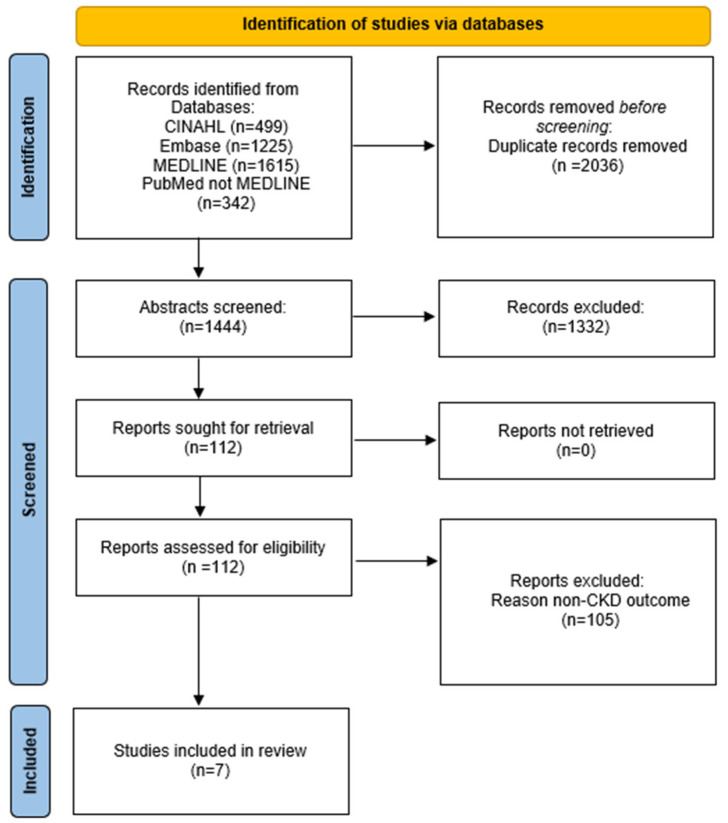
PRISMA diagram: study search criteria and selection process.

Prior to the screening process, duplicate citations were removed. Titles and abstracts were screened by two independent investigators (KEL and CP) using Covidence [19], a web-based collaboration software platform that streamlines the production of systematic and other literature reviews. Full-text articles that met our inclusion criteria were retrieved and screened by two investigators (KEL and CP) according to the study eligibility criteria presented in Table 1. Disagreements between investigators were adjudicated by group consensus. Figure 1 presents the study search criteria and selection process.

Studies were considered if they were retrospective or prospective cohort studies, cross-sectional studies, or case–control studies without a duration restriction. Eligible populations included adults (≥20 years old) residing in any country. The intervention or exposure of interest was UPF, or highly processed food intake described as group 4 of the NOVA food classification system, including studies that retrospectively applied the NOVA classification to available dietary data [7]. The outcomes of interest were CKD incidence, prevalence, and disease progression, as measured by change in estimated glomerular filtration rate (eGFR).

### 2.4. Data Extraction

Two investigators (KEL and CP) independently extracted and collected information using data extraction tables to capture study and participant characteristics as well as quantitative results focusing on measured associations between UPF intake and CKD. Study characteristics included population cohort (as available) and age, location, and design. We extracted data for the exposure UPF definition based on the NOVA classification, a dietary assessment method, and the outcome, an ascertainment of CKD.

### 2.5. Risk of Bias

Two investigators (KEL and CP) independently performed risk-of bias (RoB) assessments using the Nutrition Quality Evaluation Strengthening Tools (NUQUEST) [20]. NUQUEST focuses on the extent to which nutrition studies are designed, conducted, analyzed, and reported, though it is not intended to quantify the magnitude of bias that may be attributed to methodological flaws. A study was rated as “good” if nearly all domains showed low risk of bias, meaning that minimal or no methodological concerns were identified across key areas. A “neutral” rating was assigned when some domains (typically one to two domains) exhibited methodological concerns, resulting in a moderate risk of bias. A “poor” rating indicated that multiple domains (three or more) showed substantial methodological weaknesses, corresponding to a high risk of bias. This domain-based approach ensured a consistent and transparent evaluation of bias across studies [18].

### 2.6. Statistical Analysis

Log-transformed values of effect size were obtained from hazard ratios (HRs), and related 95% CIs were extracted for the association between UPF intake and risk of CKD. A random-effects model was used to assess the pooled risk estimates [21]. Cochrane’s Q test and *I*^2^ were considered to assess the potential sources of heterogeneity among the included studies, where *p* ≤ 0.1 was considered significant heterogeneity and I^2^ values of 25%, 50%, and 75% were interpreted as low, moderate, and high heterogeneity, respectively [22]. All data analysis and data visualizations were performed using RStudio 2023.12.1.402. (RStudio: Integrated Development Environment for R. Posit Software, PBC, Boston, MA, USA).

## 3. Results

Figure 1 presents the study search and selection process. Altogether, 3681 citations were identified through dataset searches. After duplicates were removed, 1444 remained for screening and 1332 were excluded. We retrieved 112 full-text publications, and 7 articles met the systematic review inclusion criteria [23,24,25,26,27,28,29]. The 105 excluded articles and exclusion reasons are presented in Supplementary Table S1.

### 3.1. Study Characteristics

A summary of the characteristics of all included studies is present in Table 2. All studies were published between 2021 and 2023, and none of the studies were funded by a company involved in the production of UPF, nor did any of the authors report conflicts of interest related to such a company. Most of the studies included in this review were cohort studies and most individuals were free of kidney disease at baseline. In general, different cohorts from countries around the world were used to evaluate the association between UPF intake and CKD. Included studies were conducted in the United States [25,30], the United Kingdom [27,28], Spain [23], the Netherlands [24], China [27], and Korea [26]. Studies included both men and women, but none specially evaluated differences in risk of CKD and UPF intake for men versus women [23,24,25,26,27,28,30]. Sample sizes ranged from 632 to 153,985 and follow-up time varied between 2.6 and 32 years, and retrospectively evaluated dietary data were from as early as 1987.

### 3.2. Exposure Definition

The methodology used to collect dietary data varied between the studies. Included studies used food frequency questionnaires (FFQs) at baseline only [24,27], an FFQ and 24 h recall at baseline only [27], an FFQ at baseline and one additional undefined timepoint [25], an FFQ at baseline and again at 2- and 7-year follow-up visits [30], 24 h recall at baseline only [28], and finally, a diet history at baseline only [23]. Dietary data in all studies were classified using the NOVA classification system. Participants were then grouped into quartiles or tertiles based on the proportion of energy from UPF consumption, or total energy intake was analyzed as a continuous variable. A diet score was used as an indicator of overall diet quality, though the specific scoring systems varied across studies and included the Mediterranean diet score (MDS) [24], Healthy Eating Index (HEI) [30], and Alternative Health Eating Index (AHEI) [25].

### 3.3. Outcome Definition

In this study, we aimed to conduct a systematic review to summarize available evidence on the impact of UPF on CKD risk or CKD progression. The studies identified in this review reported a total of 19,645 incident CKD cases from six cohorts of individuals free of baseline CKD. One study evaluated the risk of CKD progression, identifying 1047 cases [30]. While the studies that met our inclusion criteria assessed the association between UPF and adverse kidney outcomes, there was significant heterogeneity in the ascertainment of kidney disease outcome despite five of seven (71%) defining CKD as an eGFR < 60 mL/min/1.73 m^2^. Most of the studies used single baseline creatinine measurements to estimate kidney function and used the 2009 CKD-EPI equation for calculation.

Some studies used composite outcomes, though definitions varied. In some cases, the composite was defined as either a ≥30% decline in eGFR from baseline or incident CKD, identified by a new eGFR < 60 mL/min/1.73 m^2^ at follow-up compared with baseline. Other definitions included reduced kidney function (eGFR < 60 mL/min/1.73 m^2^) accompanied by a ≥25% decline from baseline at any follow-up visit; hospitalization with a diagnosis of CKD stage 3 or higher based on ICD-9/10 codes; death involving CKD stage 3 or higher identified via the National Death Index; or progression to end-stage kidney disease, defined as dialysis or transplantation, identified through linkage with the US Renal Data System (USRDS) [25].

All studies reported adjusted risk estimates along with 95% CIs regarding the relationship between UPF intake and CKD risk. Across the included studies, we found that there was positive and significant association that varied between 4–74% higher risk of incident CKD with increased UPF intake [23,24,25,26,27,28] and a 17–22% higher risk of CKD progression UPF intake [24,30]. All of the studies adjusted for total energy intake, and most also accounted for markers of socioeconomic status, such as income and/or education level [24,25,26,27,30]. Details of the adjustments made in the statistical analysis from the original research are provided in Table 2.

### 3.4. Risk of Bias

RoB assessments were conducted on the six cohort studies, and results are presented in Supplementary Table S2. Across all, there was moderate vulnerability to bias where two out of six studies (33%) were considered “good” while four were considered “neutral” according to the NUQUEST scale. This is largely due to the nutrition-specific domain and specifically due to the likelihood of baseline exposure maintained over the course of the follow-up period. A NUQUEST tool does not currently exist to assess biases in cross-sectional studies, so bias could not be evaluated in one study.

### 3.5. Meta-Analysis Findings

**Figure 2:**
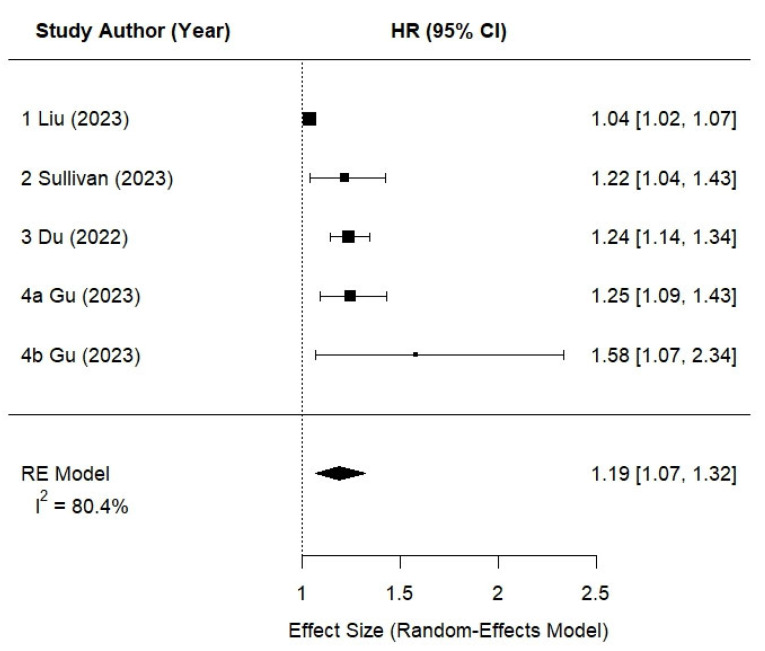
Forest plot of the meta-analysis [25,27,28,30]. Additional study details are provided in Table 2.

Studies that reported hazard ratios using categorical definitions of UPF consumption were included in the random-effects model to allow for uniform synthesis of risk estimates [25,27,28,30]. The study by Gu et al. [27] reported risk estimates separately for subgroups, and thus six risk estimates were entered into the final analysis, representing 297,387 participants. The results for the overall analysis are shown in Figure 2. Higher UPF intake was significantly associated with increased risk of CKD, with a pooled hazard ratio (HR) of 1.19 (95% CI: 1.07–1.32; *p* < 0.001). The corresponding log-HR (0.17; 95% CI: 0.07–0.28) is reported as the effect size used in the meta-analytic model, as this is the scale on which hazard ratios are statistically pooled. Between-study heterogeneity was substantial (I^2^ = 80.4%, *p* < 0.001), supporting the use of a random-effects model.

## 4. Discussion

In this current meta-analysis of seven studies using six cohorts, we report a higher risk of incident CKD and more rapid kidney function decline with increasing levels of UPF intake. There is broad generalizability of these findings given the cohorts are from The Netherlands, Spain, China, Korea, UK, and USA, highlighting shifts in traditional eating habits toward UPF dominance of the diet. The relationship between UPF and CKD remained significant after adjusting for diet quality. These findings align with the existing body of evidence that suggests that diet quality is a critical determinant of kidney health [31].

UPFs are predominantly made from substances extracted from foods, derived from food constituents, or synthesized in laboratories from food substrates or other organic sources. These substances often include oils, fats, sugars, starch, and proteins. UPFs are characterized by minimal whole food content and are frequently enhanced with additives like artificial flavors, colors, emulsifiers, and preservatives to improve sensory qualities, shelf life, and palatability [10]. Several studies have indicated that higher intake of UPF is associated with various adverse health outcomes [11].

In addition to low nutritional quality and high energy density, potential mechanisms by which UPF contributes to non-communicable diseases, including CKD, continue to be investigated. Several studies have reported the association between higher intake of sugar and sugar-sweetened beverages and risk of CKD [32,33]. Higher consumption of UPF was also related to higher intake of sugar-sweetened beverages [34]. Another potential explanation may be the effect of a high-sodium diet and increasing oxidative stress and causing alterations in the kidney and vascular systems [35,36]. Advanced glycation end products (AGEs) can also contribute to an increase in oxidative stress and inflammation, causing impairment to intestinal barrier permeability and complement pathway activation. UPFs commonly contain inorganic phosphorus additives, which are highly bioavailable [37] and problematic to those with CKD given the association with adverse kidney outcomes [38].

It is currently unclear how food additives used during processing impact kidney health. An evolving tool in nutritional epidemiology that may improve the ability to minimize the bias of traditional dietary assessment tools and provide objective measurement of dietary patterns is the discovery of dietary metabolites. A recent secondary analysis of a domiciled randomized crossover feeding trial aimed to identify metabolites that differ between dietary patterns high in or free of UPF [39]. It was reported that UPF consumption in generally healthy adults had an impact on numerous plasma and urine metabolites, suggesting their deleterious effect on the human metabolome, justifying further dietary biomarker research. Specific to CKD, Su et al. [34] sought to identify serum metabolomic biomarkers of UPF and investigate their prospective association with CKD risk. Twelve metabolites were associated with UPF, and glucose, mannose, and N2, N2-dimethylcuanosine were associated with CKD risk [34]. These results should be interpreted with caution considering the dietary assessment was completed in the late 1980s and is unlikely to accurately represent today’s intake of UPF. Nevertheless, additional research on the identification of dietary metabolites’ association with UPF intake and their association with CKD risk is warranted.

There are multiple food classification systems that have been developed, classifying foods on the basis of processing level [40]. The NOVA classification system is the most often used in epidemiological research despite criticism of its lack of rigorous definitions for processing categories, undefined cutoff values for food additives and nutrients, and coding methodologies that change over time [41]. While the impact of processing and potential misclassification will greatly impact conclusions related to disease outcomes, we would like to additionally highlight the numerous dietary intake methodologies used to capture dietary intake among the cohorts in this review and implications for result interpretation. Dietary assessment tools such as 24 h recalls, food frequency questionnaires, and diet dairies may result in random and systematic errors originating from daily variations in individual food choices, not capturing the entire diet, recall difficulty, or reporting errors [42]. Total calorie intake and protein are generally underreported using all of these methods; however, given the prospective cohort design of most included studies, it may be appropriate to advance the understanding of dietary intakes and the relationship between diet and health [42].

In nearly all the included studies, dietary assessment was measured at baseline and was not repeated during the length of follow-up. This makes it difficult to rule out any reverse causality that may have occurred with a participant being informed of declining kidney function as well as the interpretation of the longitudinal effect of UPF on kidney function. It is accepted in guideline documents that diet modification, through a Mediterranean dietary pattern, may improve lipid profiles and increasing the consumption of fruits and vegetables may decrease body weight, blood pressure, and net acid production, all predictors of disease progression [38]. A recent analysis of the ARIC cohort found that greater adherence to healthy dietary patterns, measured by the HEI-2015, AHEI-2010, and aMed scores, was associated with a lower risk of incident CKD [43]. This suggests that healthcare providers are likely to offer dietary guidance following a CKD diagnosis, which would not be captured without repeated dietary assessments.

For this reason, we recommend that future studies include multiple 24 h recalls and an FFQ, validated for the particular population, on all participants. This approach provides the most flexibility for analysis and combines the accuracy of the 24 h recall with the ability to capture additional information on less frequently consumed foods/beverages from the FFQ.

Capturing multiple dietary assessments would also allow for enhanced understanding of the lagged effects of UPF and CKD risk. With longer latency between diet and outcome assessment, the analyses may be conservative though would exclude the effect of reverse causation. A recent publication found that diet has more immediate effects on the prevention of major cardiometabolic disease such as coronary heart disease and stroke as well as type 2 diabetes [44]. However, a longer latency period was required for major types of cancer. Given the slow and progressive nature of CKD [45], it may be prudent to capture the full lifetime disease association of UPF intake among younger individuals in addition to midlife/older adults. Analyses reviewed here were completed on midlife adults, so we are unable to capture the cumulative exposure to UPF and CKD risk and therefore suggest that future studies include younger populations in their cohorts.

Dietary information from the included studies was captured over the course of three decades from cohorts in six different countries. While the NOVA classification was applied, we would like to highlight the significant change in food consumption and differing food choices from each country during this time period. A recent publication reported that the change in UPF intake among a nationally representative sample of US adults increased by 3.5% over the last 18 years [46]. Intake from around the world is following similar alarming trends, with the highest intakes from North America, Europe, Australia, and Latin America and rapidly growing sales in the Middle East, Asia, and Africa [47]. Even though the regional differences in intake may be minimized by consistent use of the NOVA classification system [5], it is likely that the drastic differences in food consumption patterns over time do not accurately estimate the true association between UPF intake and risk of incident CKD.

We would also like to highlight the heterogeneity in CKD definitions and ascertainment methods used across the studies included, which limits the comparability of the results. Glomerular filtration rate is estimated (eGFR) from serum concentrations of endogenous markers including creatinine or cystatin C and is used as a guide for clinical decision-making [48]. eGFR slope change has shown a strong association with clinical endpoints, including death or initiation of kidney replacement therapy, and thus is accepted as a surrogate endpoint for CKD progression in clinical trials [49]. Various approaches to calculating eGFR exist, and thus estimates and clinical care decisions are not standardized, leading to disparities in care. Previous studies indicated a higher average serum creatinine level for the same measured GFR level in Black participants than in non-Black participants [50,51]. New creatinine-based GFR equations without race were proposed and are considered sufficiently accurate for clinical practice [52]. Creatinine and cystatin C equations without race more accurately estimated measured GFR and led to smaller differences between race groups. In addition to varying methods to define CKD, the length of follow-up time varied between 2 and 32 years, respectively, and repeated kidney function assessments were not always completed. Identification of short-term eGFR decline in these cohort studies could provide useful insight into the association of diet and disease progression. A recent consensus defined a reduction in slope of eGFR decline by 0.75 mL/min per 1.73 m^2^ per year over 2 years as being associated with a 30% lower risk of subsequent end-stage kidney disease [53]. Therefore, we recommend the inclusion of cystatin C in the biometric evaluation of cohorts and repeated eGFR calculations to calculate the slope, as a rapid decrease in eGFR over the short term is strongly and consistently linked with an increased likelihood of developing end-stage kidney disease later on.

Adherence to the Mediterranean and DASH dietary patterns leads to better kidney function outcomes following transplantation [54], and overall guidance is to reduce comorbidities and micronutrient deficiencies post-transplant [55]. There is a gap in knowledge regarding the effect of UPF and mortality among kidney transplant recipients, and Osté et al. [56] reported that higher intake of UPF was associated with two times the risk of mortality, with significant associations identified between sugar-sweetened beverages, desserts, and processed meats. Similar connections to mortality have been reported among generally healthy, diverse populations [57,58], and one specifically reported that greater than four servings of UPF per day was associated with increased mortality risk [59]. Overall poor diet quality and exposure to chemical additives, colorants, and flavorings may be contributing factors to the associations described. Longitudinal research is essential to determine the extended consequences of UPF consumption on overall mortality, shedding light on how dietary habits influence health over time and identify critical periods where dietary intake could have the greatest impact.

Dietary intake plays a significant role in the development and progression of CKD, yet nearly half of individuals with CKD do not follow a healthy diet [29]. Diet is just one aspect of a healthy lifestyle. Other factors including physical activity, healthy weight management, and limited tobacco use and alcohol consumption are part of various guideline recommendations [60]. Other factors that need to be considered when assessing the relationship between diet and CKD include sociodemographic and economic factors (e.g., sex, race/ethnicity, and access to nephrology care), genetic factors (e.g., APOL1 genotype), cardiovascular factors (e.g., atrial fibrillation, hypertension, and vascular stiffness), cardiometabolic disease (e.g., diabetes and obesity), and metabolic factors (e.g., FGF23 and urinary oxalate) [61]. These variables potentially confound or distort the true association between diet and CKD risk. In order to control for these factors, multivariable analysis is required to disentangle the individual contribution of each predictor while controlling for the influence of the other factors. When evaluating the studies that met our inclusion criteria, there was significant heterogeneity in variable types, variable measurement, modeling impact, and effect size, rendering it difficult to provide a comparative context within the broader research landscape. It is imperative that while ensuring comparability in essential dimensions across each cohort, we also harness their distinctive characteristics to enrich the literature, addressing not only the impact of UFP consumption on CKD risk but also the broader nexus between dietary patterns and CKD susceptibility.

### Limitations

In addition to the factors discussed previously, the results of the meta-analysis must be interpreted with caution. This meta-analysis was based on available cohort studies and not randomized trials, so the findings should not be interpreted as causative. Original research using the NOVA classification system to assess food processing levels was completed in Brazil, and we limited our inclusion criteria to publications available in English, excluding potential relevant non-English sources. The NOVA classification system utilizes an expertise-driven manual evaluation of each food and placement into broad, mutually exclusive categories that may not capture the full complexity of food processing. A recent proof-of-concept study reported that a menu composed of nearly all calories from UPF can align with the Dietary Guideline for Americans recommendations and contain adequate amounts of most macro and micro nutrients [62], suggesting it is possible to observe dietary patterns consisting primarily of UPF and evaluate and compare the utility of different classification systems.

It is important to note that the widespread availability and accessibility of these foods in various regions around the world may be limited, and they are more easily found in major metropolitan areas in the United States. Efforts should be made to improve the availability and accessibility of these foods in regions where they are currently limited, ensuring that populations outside major metropolitan areas also have access. Nevertheless, this calls into question the usefulness of broad categorization and perhaps greater attention should be placed on the degree of food processing, amount of non-culinary additives, and utilization of machine learning to predict the degree of processing [63]. To examine the relationship between a dietary pattern and CKD, proper adjustment for confounding variables including energy intake, baseline kidney function, history of hypertension and/or diabetes, and socio-economic status should be included in the models. Participants were from a diverse population, though specific information on race was insufficient to assess the potential effect of race and diet on the observed outcomes. Additionally, because the number of studies included in the meta-analysis was relatively small, we were unable to formally assess publication bias, and the possibility of publication bias cannot be excluded. Results may not be generalizable to all populations around the world.

## 5. Conclusions

Overall, available evidence reviewed in this study suggests there is an association between higher UPF intake and CKD risk. The significant heterogeneity in dietary assessment methodology, temporal availability of dietary information, and ascertainment methodology of CKD warrants caution when interpreting these results. Additional studies using more sophisticated classification techniques to improve the precision of harmful UPF identification and understanding how different foods contribute to dietary health risks are required to identify whether the association between a dietary pattern higher in UPF and risk of incident kidney disease could be clinically useful to decrease the risk of this health outcome and be included in nutrition-related guidelines.

## Figures and Tables

**Table 1:** Search criteria.

Category	Inclusion Criteria	Exclusion Criteria
Study Design	Any study including the following: Retrospective cohorts; Prospective cohorts; Cross-sectional studies; Case–control studies.	Narrative reviews Systematic reviews Meta-analysis Letters to the editor Conference proceedings Abstracts
Study Duration	No restriction	No restriction
Sample Size	No restriction	Studies with insufficient reporting outcomes
Intervention/Exposure	Ultra-processed food or highly processed	Studies assessing only unprocessed, minimally processed, or other food exposures without UPF focus
Comparator	None	None
Outcomes	Chronic kidney disease (CKD) incidence, prevalence, and disease progression as measured by change in eGFR	Studies reporting only non-CKD outcomes
Date of Publication	After 1 January 2009	Prior to 1 January 2009
Publication Status	Article published in peer-reviewed journals	Non-peer-reviewed sources, unpublished studies, conference abstracts
Language of Publication	English	Languages other than English
Country	No restriction	No restriction
Study Participants	Human subjects	Studies on non-human subjects, pediatric populations (participants ≤ 19 years old), or exclusively gestational outcomes (e.g., pregnancy-specific kidney outcomes)
Age of Study Participants	Adults ≥ 20 years (based on mean/median if available or mid-point of reported age range)	Participants ≤ 19 years (based on mean/median if available or mid-point of reported age range)

**Table 2:** Study characteristics of included studies.

Publication Details	Sample Description (Size, Age, Length of Follow-Up, Cohort, Location)	Dietary Assessment	Kidney Function	Adjustment	Comparison	OR, RR, or HR (95% CI)
1. Liu et al., 2023 [28]	N = 153,985, age (mean): 55.9 ± 8.0 years, follow-up (median): 12.1 years, UK Biobank, UK	24 h recall [baseline]	Self-report data and data linkage with primary care, hospital admissions, and death registry records based on the International Classification of Diseases, 10th revision (ICD-10) coding system	Adjusted for age, sex, race, Townsend Deprivation Index, body mass index, systolic and diastolic blood pressure, history of hypertension, high cholesterol, smoking status, alcohol consumption, physical activity, healthy diet score, total energy, c-reactive protein, eGFR, urine albumin/creatinine ratio	T3 vs. T1	per 10% increment, adjusted HR: 1.04; (1.01; 1.06) [total population] adjusted HR: 1.11; (1.05; 1.17) [with diabetes] adjusted HR: 1.03; (1.00; 1.05) [without diabetes]
2. Sullivan et al., 2023 [30]	N = 2616, age: (mean) 58 ± 11 years, follow-up (median): 7 years, CRIC, USA	FFQ [baseline, 2, 7-year follow-up]	≥50% decrease in eGFR or initiation of kidney replacement therapy [2021 CKD-EPI equation without race]	Adjusted for age, sex, race, total energy intake, education, income, smoking status, physical activity, study site, eGFR, proteinuria, body mass index, systolic blood pressure, number of blood pressure medications, diabetic status, antiplatelet medication use, lipid-lowering medication, Healthy Eating Index-2015 score	T3 vs. T1	HR: 1.22 (1.04–1.42) *p* = 0.01
3. Du et al., 2022 [25]	N = 14,679, age: 45–64, follow-up (median): 32 (24) years, ARIC Cohort, USA	FFQ [baseline (1987–1989) and visit 3 (1993–1995)]	(1) reduced kidney function (eGFR < 60 mL/min/1.73 m^2^) accompanied by ≥25% eGFR decline at any follow-up study visit relative to baseline; (2) hospitalization involving CKD stage 3+ diagnosis defined by International Classification of Diseases (ICD) 9/10 code, identified through active surveillance of the ARIC cohort; (3) death involving CKD stage 3+ diagnosis defined by ICD 9/10 code, identified through linkage to the National Death Index; or (4) end-stage kidney disease defined as dialysis or transplantation, identified by linkage to the USRDS registry	Adjusted for age, sex, race, total energy intake, education level, smoking status, physical activity score	Q4 vs. Q1	Visit-based definition HR: 1.22, (1.09, 1.37) *p* trend = 0.009 Composite-based definition HR: 1.19 (1.09, 1.29) *p* < 0.0001
4a. Gu et al., 2023 [27]	N = 23,775, age (mean): 33.6–47.5 years, follow-up (median): 4 years, TCLSIH cohort, Tianjin China	FFQ [baseline]	eGFR < 60 mL/min/1.73 m^2^, albumin-to-creatinine ratio 30 mg/g, or as having a clinical diagnosis of CKD [MDRD study equation]	Adjusted for age, sex, education level, employment status, household income, body mass index, smoking status, alcohol drinking status, physical activity, dietary pattern, total energy intake, family history of hypertension, cardiovascular disease, hyperlipidemia, diabetes, other kidney disease, high-sensitivity C-reactive protein, albumin, eGFR	Q4 vs. Q1	HR: 1.58 (1.07, 2.34) *p* = 0.02
4b. Gu et al., 2023 [27]	N = 102,332, age (mean): 55–58, follow-up (median): 10.1 years, UK Biobank, UK	24 h recall [baseline]	eGFR < 60 mL/min/1.73 m^2^ or as having a clinical diagnosis of CKD, which was ascertained based on information from medical and death records. [MDRD study equation] Incident CKD was ascertained based on information from medical and death records	Adjusted for age, sex, education level, Townsend deprivation index, body mass index, smoking status, alcohol drinking status, physical activity, healthy dietary score, total energy intake, family history of hypertension, cardiovascular disease, diabetes, other kidney disease, high-sensitivity C-reactive protein, eGFR	Q4 vs. Q1	HR: 1.25 (1.09, 1.43) *p* < 0.001
5. Cai et al., 2022 [24]	N = 78,346, age: 45.8 ± 12.6, mean follow-up 3.6 ± 0.9 years, Lifelines Cohort, Netherlands	FFQ [baseline] (2006–2011)	Composite outcome [≥ 30% eGFR decline or incident CKD (<60 mL/min/1.73 m^2^)] at the second study visit [2009 CKD-EPI equation]	Adjusted for age, sex, baseline eGFR, history of diabetes, hypertension, or cardiovascular disease, physical activity, smoking total energy intake, education level, Mediterranean diet score, energy-adjusted protein, carbohydrate and fat intake	Q4 vs. Q1	OR: 1.27 (1.09–1.47) *p* = 0.003 Highest quartile had more rapid eGFR decline (β, −0.17; 95% CI, −0.23 to −0.11; *p* < 0.001)
6. Rey-Garcia et al., 2021 [23]	N = 1312, age: 67 ± 5.5 years, follow-up: 6 years, Seniors-ENRICA-1, Spain	Diet history [baseline] 2008–2010	SCr increased or an eGFR decreased beyond that expected for age. Change in eGFR beyond that expected for age was calculated in 3 steps: (i) eGFR based on baseline creatinine and age in 2015; (ii) eGFR in 2015 based on both SCr and eGFR in 2015; and (iii) subtracting ii from i [2009 CKD-EPI equation]	Adjusted for age, sex, total energy intake, education level, smoking status, drinking status, physical activity, time spent watching television, total fiber intake, number of chronic conditions, number of medications, history of hypertension, diabetes, hypercholesterolemia, body mass index	T3 vs. T1	OR: 1.74 (1.14–2.66) *p* = 0.026
7. Kityo et al., 2022 [26]	N = 134,544, age (mean): 52 years, follow-up: N/A, HEXA cohort, Korea	FFQ [baseline]	eGFR < 60 mL/min/1.73 m^2^ [2009 CKD-EPI equation]	Adjusted for age, sex, total energy intake, education level, income, smoking, drinking status, physical activity, body mass index, history of hypertension, high blood sugar, prevalent cardiovascular disease	Q4 vs. Q1	PR: 1.16 (1.07, 1.25) *p* = 0.003

HR: hazard ratio; UK: United Kingdom; CRIC: Chronic Renal Insufficiency Cohort; USA: United States of America; FFQ: Food Frequency Questionnaire; CKD: chronic kidney disease; ARIC: Atherosclerosis Risk in Communities Study; eGFR: estimated glomerular filtration rate; TCLSIH: Tianjin Chronic Low-Grade Systemic Inflammation and Health; USRDS: United States Renal Data System; ENRICA-1: Nutrition and Cardiovascular Risk in Spain; Scr: serum creatinine; T: tertile; Q: quartile; OR: odds ratio; HEXA: Health Examinees Study; PR: prevalence ratio.

## Supplementary Materials

The following supporting information can be downloaded at: https://www.mdpi.com/article/10.3390/nu17091560/s1, Table S1: Publications excluded from the review with reasons for exclusion; Table S2: Risk of Bias assessment using NUQUEST Tool.
