# Supplementary material for: Ultra-Processed Food and Chronic Kidney Disease Risk: A Systematic Review, Meta-Analysis, and Recommendations
Source: Nutrients. 2025 Apr 30;17(9):1560. doi: 10.3390/nu17091560 (PMC12073181; doi:10.3390/nu17091560)
Supplement: Supplementary file 1 [file nutrients-17-01560-s001.zip › nutrients-3615939 Supplementary Materials.pdf]

Supplementary Materials:

Supplemental Table S1. Publications excluded from the review with reasons for exclusion

First Author	Year	Title	Reason for Excluding
Anonymous [1]	2018	Scientists Find Link Between Cancer and Highly Processed Foods	Non-CKD outcome
Arvanitakis [2]	2023	We Are What We Eat: The Association Between Ultra-Processed Food Consumption, Colon Cancer Risk and All-Cause Mortality	Non-CKD outcome
Atzeni [3]	2022	Association between ultra-processed food consumption and gut microbiota in senior subjects with overweight/obesity and metabolic syndrome	Non-CKD outcome
Barbosa [4]	2023	Ultra-processed food consumption and metabolic syndrome: a cross-sectional study in Quilombola communities of Alagoas, Brazil	Non-CKD outcome
Beslay [5]	2020	Ultra-processed food intake in association with BMI change and risk of overweight and obesity: A prospective analysis of the French NutriNet-Santé cohort	Non-CKD outcome
Blanco-Rojo [6]	2019	Consumption of Ultra-Processed Foods and Mortality: A National Prospective Cohort in Spain	Non-CKD outcome
Bonaccio [7]	2021	Ultra-processed food consumption is associated with increased risk of all-cause and cardiovascular mortality in the Moli-sani Study	Non-CKD outcome
Bonaccio [8]	2022	Joint association of food nutritional profile by Nutri-Score front-of-pack label and ultra-processed food intake with mortality: Moli-sani prospective cohort study	Non-CKD outcome
Bonaccio [9]	2022	Ultra-processed Food Intake and All-cause and Cause-specific Mortality in Subjects with Type 2 Diabetes: Longitudinal Findings from the Moli-sani Study	Non-CKD outcome
Bonaccio [10]	2022	Ultra-processed food intake and all-cause and cause-specific mortality in individuals with cardiovascular disease: the Moli-sani Study	Non-CKD outcome
Bonaccio [11]	2023	Ultraprocessed food consumption is associated with all-cause and cardiovascular mortality in participants with type 2 diabetes independent of diet quality: a prospective observational cohort study	Non-CKD outcome
Canella [12]	2014	Ultra-processed food products and obesity in Brazilian households (2008-2009)	Non-CKD outcome
Canhada [13]	2020	Ultra-processed foods, incident overweight and obesity, and longitudinal changes in weight and waist circumference: the Brazilian Longitudinal Study of Adult Health (ELSA-Brasil)	Non-CKD outcome

Canhada [14]	2023	Ultra-Processed Food Consumption and Increased Risk of Metabolic Syndrome in Adults: The ELSA-Brasil	Non-CKD outcome
Cardoso [15]	2022	Association between ultra-processed food consumption and cognitive performance in US older adults: a cross-sectional analysis of the NHANES 2011-2014	Non-CKD outcome
Chang [16]	2023	Ultra-processed food consumption, cancer risk and cancer mortality: a large-scale prospective analysis within the UK Biobank	Non-CKD outcome
Chen [17]	2022	Associations of ultra-processed food consumption with cardiovascular disease and all-cause mortality: UK Biobank	Non-CKD outcome
Chen [18]	2023	Intake of Ultra-processed Foods Is Associated with an Increased Risk of Crohn's Disease: A Cross-sectional and Prospective Analysis of 187 154 Participants in the UK Biobank	Non-CKD outcome
Cordova [19]	2021	Consumption of ultra-processed foods associated with weight gain and obesity in adults: A multi-national cohort study	Non-CKD outcome
Cross [20]	2023	Ultra-processed foods and colorectal neoplasia: is there a link?	Non-CKD outcome
da Silva [21]	2021	Ultra-processed foods consumption is associated with cardiovascular disease and cardiometabolic risk factors in Brazilians with established cardiovascular events	Non-CKD outcome
da Silva [22]	2021	Usual consumption of ultra-processed foods and its association with sex, age, physical activity, and body mass index in adults living in Brasília city, Brazil	Non-CKD outcome
da Silva [23]	2022	Association of Food Intake with Sleep Durations in Adolescents from a Capital City in Northeastern Brazil	Non-CKD outcome
Detopoulou [24]	2023	Dietary acid load is associated with waist circumference in university students with low adherence to the Mediterranean diet: The potential role of ultra-processed foods	Non-CKD outcome
Detopoulou [25]	2023	Relation of Minimally Processed Foods and Ultra-Processed Foods with the Mediterranean Diet Score, Time-Related Meal Patterns and Waist Circumference: Results from a Cross-Sectional Study in University Students	Non-CKD outcome
Dehghan [26]	2023	Ultra-processed foods and mortality: analysis from the Prospective Urban and Rural Epidemiology study	Non-CKD outcome
Donat-Vargas [27]	2021	High Consumption of Ultra-Processed Food is Associated with Incident Dyslipidemia: A Prospective Study of Older Adults	Non-CKD outcome

Du [28]	2021	Higher Ultra-Processed Food Consumption Is Associated with Increased Risk of Incident Coronary Artery Disease in the Atherosclerosis Risk in Communities Study	Non-CKD outcome
Duan [29]	2022	Ultra-processed food and incident type 2 diabetes: studying the underlying consumption patterns to unravel the health effects of this heterogeneous food category in the prospective Lifelines cohort	Non-CKD outcome
El Kinany [30]	2022	Food processing groups and colorectal cancer risk in Morocco: evidence from a nationally representative case-control study	Non-CKD outcome
Esposito [31]	2023	Food processing and risk of central nervous system tumours: A preliminary case-control analysis from the MEditerranean DIet in relation to CancEr of brAin (MEDICEA) study	Non-CKD outcome
Fiolet [32]	2018	Consumption of ultra-processed foods and cancer risk: results from NutriNet-Sante prospective cohort	Non-CKD outcome
Fitzpatrick [33]	2023	Ultra-processed Foods and Risk of Crohn's Disease: How Much is Too Much?	Non-CKD outcome
Godos [34]	2023	Ultra-Processed Food Consumption and Depressive Symptoms in a Mediterranean Cohort	Non-CKD outcome
Gomez-Donoso [35]	2020	Ultra-processed food consumption and the incidence of depression in a Mediterranean cohort: the SUN Project	Non-CKD outcome
Gourd [36]	2018	Ultra-processed foods might increase cancer risk	Non-CKD outcome
Haghighatdoost [37]	2022	The relationship between ultraprocessed food consumption and obesity indicators in Iranian adults	Non-CKD outcome
Hall [38]	2019	Ultra-Processed Diets Cause Excess Calorie Intake and Weight Gain: An Inpatient Randomized Controlled Trial of Ad Libitum Food Intake	Non-CKD outcome
Hang [39]	2023	Ultra-processed food consumption and risk of colorectal cancer precursors: results from 3 prospective cohorts	Non-CKD outcome
Hosseininasab [40]	2022	The relationship between ultra-processed food intake and cardiometabolic risk factors in overweight and obese women: A cross-sectional study	Non-CKD outcome
Ivancovsky-Wajcman [41]	2021	Ultra-processed food is associated with features of metabolic syndrome and non-alcoholic fatty liver disease	Non-CKD outcome
Jacobs [42]	2022	Degree of food processing and breast cancer risk in black urban women from Soweto, South African: the South African Breast Cancer study	Non-CKD outcome
Jafari [43]	2023	Ultra-Processed Food Intake and Risk of Colorectal Cancer: A Matched Case-Control Study	Non-CKD outcome
Juul [44]	2018	Ultra-processed food consumption and excess weight among US adults	Non-CKD outcome

Juul [45]	2021	Ultra-Processed Foods and Incident Cardiovascular Disease in the Framingham Offspring Study	Non-CKD outcome
Kim [46]	2019	Ultra-processed food intake and mortality in the USA: results from the Third National Health and Nutrition Examination Survey (NHANES III, 1988-1994)	Non-CKD outcome
Kim [47]	2023	Relationship between metabolic syndrome and intake of ultra-processed foods in Korean adults: based on 6th and 7th Korea National Health and Nutrition Examination Survey (2013-2018)	Non-CKD outcome
Kityo [48]	2023	The intake of ultra-processed foods, all-cause, cancer and cardiovascular mortality in the Korean Genome and Epidemiology Study-Health Examinees (KoGES-HEXA) cohort	Non-CKD outcome
Kliemann [49]	2023	Food processing and cancer risk in Europe: results from the prospective EPIC cohort study	Non-CKD outcome
Konieczna [50]	2021	Contribution of ultra-processed foods in visceral fat deposition and other adiposity indicators: Prospective analysis nested in the PREDIMED-Plus trial	Non-CKD outcome
Lee [51]	2022	Ultra-Processed Foods as a Less-Known Risk Factor in Cardiovascular Diseases	Non-CKD outcome
Levy [52]	2021	Ultra-processed food consumption and type 2 diabetes incidence: A prospective cohort study	Non-CKD outcome
Li [53]	2021	Ultra-Processed Food Consumption Associated with Overweight/Obesity among Chinese Adults-Results from China Health and Nutrition Survey 1997-2011	Non-CKD outcome
Li [54]	2023	Association of Ultra-Processed Food Intake with Cardiovascular and Respiratory Disease Multimorbidity: A Prospective Cohort Study	Non-CKD outcome
Liu [55]	2023	Association between ultra-processed foods consumption and risk of non-alcoholic fatty liver disease: A population-based analysis of NHANES 2011-2018	Non-CKD outcome
Liu [56]	2023	Consumption of Ultraprocessed Foods and Body Fat Distribution Among U.S. Adults	Non-CKD outcome
Llavero-Valero [57]	2021	Ultra-processed foods and type-2 diabetes risk in the SUN project: A prospective cohort study	Non-CKD outcome
Lo [58]	2022	Ultra-processed Foods and Risk of Crohn's Disease and Ulcerative Colitis: A Prospective Cohort Study	Non-CKD outcome
Louzada [59]	2022	Corrigendum: Changes in Obesity Prevalence Attributable to Ultra-Processed Food Consumption in Brazil Between 2002 and 2009	Non-CKD outcome
Machado [60]	2020	Ultra-processed food consumption and obesity in the Australian adult population	Non-CKD outcome

Magalhaes [61]	2022	Sex-Dependent Effects of the Intake of NOVA Classified Ultra-Processed Foods on Syndrome Metabolic Components in Brazilian Adults	Non-CKD outcome
Martinez-Perez [62]	2021	Use of Different Food Classification Systems to Assess the Association between Ultra-Processed Food Consumption and Cardiometabolic Health in an Elderly Population with Metabolic Syndrome (PREDIMED-Plus Cohort)	Non-CKD outcome
Martinez Steele [63]	2019	Dietary share of ultra-processed foods and metabolic syndrome in the US adult population	Non-CKD outcome
Martinez Steele [64]	2022	Potential reductions in ultra-processed food consumption substantially improve population cardiometabolic-related dietary nutrient profiles in eight countries	Non-CKD outcome
Mendonca [65]	2016	Ultraprocessed food consumption and risk of overweight and obesity: the University of Navarra Follow-Up (SUN) cohort study	Non-CKD outcome
Mendonca [66]	2017	Ultra-Processed Food Consumption and the Incidence of Hypertension in a Mediterranean Cohort: The Seguimiento Universidad de Navarra Project	Non-CKD outcome
Monge [67]	2018	Ultra-processed foods and cancer	Non-CKD outcome
Monge [68]	2021	Ultraprocessed beverages and processed meats increase the incidence of hypertension in Mexican women	Non-CKD outcome
Montero Salazar [69]	2020	High consumption of ultra-processed food may double the risk of subclinical coronary atherosclerosis: the Aragon Workers' Health Study (AWHS)	Non-CKD outcome
Moreira [70]	2022	Projected impact of change in the percentage of energy from each NOVA group intake on cardiovascular disease mortality in Brazil: a modelling study	Non-CKD outcome
Mullen [71]	2021	Ultraprocessed food and cardiometabolic disease	Non-CKD outcome
Nardocci [72]	2021	Consumption of ultra-processed foods is associated with obesity, diabetes and hypertension in Canadian adults	Non-CKD outcome
Narula [73]	2021	Association of ultra-processed food intake with risk of inflammatory bowel disease: prospective cohort study	Non-CKD outcome
Nilson [74]	2022	The estimated burden of ultra-processed foods on cardiovascular disease outcomes in Brazil: A modeling study	Non-CKD outcome
Osté [75]	2022	Ultra-processed foods and risk of all-cause mortality in renal transplant recipients	Non-CKD outcome
Pang [76]	2023	Ultra-processed food consumption and obesity indicators in individuals with and without type 1	Non-CKD outcome

		diabetes mellitus: a longitudinal analysis of the prospective Coronary Artery Calcification in Type 1 Diabetes (CACTI) cohort study	
Peres [77]	2022	Consumption of processed and ultra-processed foods by patients with stomach adenocarcinoma: a multicentric case-control study in the Amazon and southeast regions of Brazil	Non-CKD outcome
Pestoni [78]	2021	Ultraprocessed Food Consumption is Strongly and Dose-Dependently Associated with Excess Body Weight in Swiss Women	Non-CKD outcome
Rauber [79]	2021	Ultra-processed food consumption and risk of obesity: a prospective cohort study of UK Biobank	Non-CKD outcome
Rezende-Alves [80]	2021	Food processing and risk of hypertension: Cohort of Universities of Minas Gerais, Brazil (CUME Project)	Non-CKD outcome
Rico-Campà [81]	2019	Association between consumption of ultra-processed foods and all-cause mortality: SUN prospective cohort study	Non-CKD outcome
Romaguera [82]	2021	Consumption of ultra-processed foods and drinks and colorectal, breast, and prostate cancer	Non-CKD outcome
Romieu [83]	2022	Consumption of industrial processed foods and risk of premenopausal breast cancer among Latin American women: the PRECAMA study	Non-CKD outcome
Sandoval-Insausti [84]	2020	Ultra-Processed Food Consumption Is Associated with Abdominal Obesity: A Prospective Cohort Study in Older Adults	Non-CKD outcome
Santos [85]	2023	Consumption of ultra- and non-ultra-processed foods of individuals with normal-weight obesity	Non-CKD outcome
Scaranni [86]	2021	Ultra-processed foods, changes in blood pressure and incidence of hypertension: the Brazilian Longitudinal Study of Adult Health (ELSA-Brasil)	Non-CKD outcome
Schnabel [87]	2018	Association Between Ultra-Processed Food Consumption and Functional Gastrointestinal Disorders: Results From the French NutriNet-Sante Cohort	Non-CKD outcome
Schnabel [88]	2019	Association Between Ultraprocessed Food Consumption<br>and Risk of Mortality Among Middle-aged Adults in France	Non-CKD outcome
Shim [89]	2022	Consumption of Ultra-Processed Food and Blood Pressure in Korean Adults	Non-CKD outcome
Shim [90]	2023	Ultra-Processed Food Consumption and Obesity in Korean Adults	Non-CKD outcome
Silva [91]	2018	Consumption of ultra-processed food and obesity: cross sectional results from the Brazilian Longitudinal Study of Adult Health (ELSA-Brasil) cohort (2008-2010)	Non-CKD outcome

Silva [92]	2023	Association of minimally processed and ultra-processed food daily consumption with obesity in overweight adults: a cross-sectional study	Non-CKD outcome
Smaira [93]	2020	Ultra-processed food consumption associates with higher cardiovascular risk in rheumatoid arthritis	Non-CKD outcome
Smiljanec [94]	2020	Associations of Ultra-Processed and Unprocessed/Minimally Processed Food Consumption with Peripheral and Central Hemodynamics, and Arterial Stiffness in Young Healthy Adults	Non-CKD outcome
Srour [95]	2019	Ultra-processed food intake and risk of cardiovascular disease: prospective cohort study (NutriNet-Sante)	Non-CKD outcome
Srour [96]	2020	Ultraprocessed Food Consumption and Risk of Type 2 Diabetes Among Participants of the NutriNet-Sante Prospective Cohort	Non-CKD outcome
Sun [97]	2023	Association of ultra-processed food consumption with incident depression and anxiety: a population-based cohort study	Non-CKD outcome
Trudeau [98]	2020	Extent of Food Processing and Risk of Prostate Cancer: The PROtEuS Study in Montreal, Canada	Non-CKD outcome
Vandevijvere [99]	2019	Global trends in ultraprocessed food and drink product sales and their association with adult body mass index trajectories	Non-CKD outcome
Vasseur [100]	2021	Dietary Patterns, Ultra-processed Food, and the Risk of Inflammatory Bowel Diseases in the NutriNet-Santé Cohort	Non-CKD outcome
Vellinga [101]	2023	Different Levels of Ultraprocessed Food and Beverage Consumption and Associations with Environmental Sustainability and All-cause Mortality in EPIC-NL	Non-CKD outcome
Yang [102]	2020	Ultra-Processed Foods and Excess Heart Age Among U.S. Adults	Non-CKD outcome
Zhang [103]	2021	Association between consumption of ultra-processed foods and hyperuricemia: TCLSIH prospective cohort study	Non-CKD outcome
Zhang [104]	2022	Ultra-processed food consumption and the risk of non-alcoholic fatty liver disease in the Tianjin Chronic Low-grade Systemic Inflammation and Health Cohort Study	Non-CKD outcome
Zhong [105]	2021	Ultra-processed food consumption and the risk of pancreatic cancer in the Prostate, Lung, Colorectal and Ovarian Cancer Screening Trial	Non-CKD outcome

Supplemental Table S2. Risk of Bias assessment using NUQUEST Tool[106]

Study Name	Study Design	Domain 1: Selection of Cohort	Domain 2: Comparability of Cohort	Domain 3: Ascertainment of Outcomes	Domain 4: Nutrition Specific	Overall Study Rating	Comments
Cai 2022	Cohort	Good	Neutral	Good	Neutral	Good	
Du 2022	Cohort	Good	Good	Good	Neutral	Good	
Gu 2023	Cohort	Good	Neutral	Neutral	Neutral	Neutral	
Kityo 2022	Cross-Sectional	N/A	N/A	N/A	N/A	N/A	Cross-sectional
Liu 2023	Cohort	Good	Good	Poor	Good	Neutral	
Rey-Garcia 2021	Cohort	Good	Good	Neutral	Good	Neutral	
Sullivan 2023	Cohort	Good	Neutral	Good	Neutral	Neutral	
NUQUEST = NUtrition QUality Evaluation Strengthening Tools							
An overall risk of bias rating was reached by following the guidance provided by the revised Cochrane risk-of-bias tool for randomized trials (RoB 2). Individual domain ratings of 'good' for all domains under assessment results in an overall rating of 'good.' Judging a result to be at a particular level of risk of bias for an individual domain (neutral or poor) implies that the result has an overall risk of bias at least this severe (neutral or poor). A NUQUEST tool does not currently exist to assess biases in cross-sectional studies.							

References

1. Anonymous. Scientists Find Link Between Cancer and Highly Processed Foods. Cancer. 2018;124(15):3079.
2. Arvanitakis M. We Are What We Eat: The Association Between Ultra-Processed Food Consumption, Colon Cancer Risk and All-Cause Mortality. Gastroenterology. 2023;164(4):697-8.
3. Atzeni A, Martinez MA, Babio N, Konstanti P, Tinahones FJ, Vioque J, et al. Association between ultra-processed food consumption and gut microbiota in senior subjects with overweight/obesity and metabolic syndrome. Front. 2022;9:976547.
4. Barbosa LB, Vasconcelos NBR, Dos Santos EA, Dos Santos TR, Ataide-Silva T, Ferreira HDS. Ultra-processed food consumption and metabolic syndrome: a cross-sectional study in Quilombola communities of Alagoas, Brazil. International Journal for Equity in Health. 2023;22(1):14.
5. Beslay M, Srour B, Mejean C, Alles B, Fiolet T, Debras C, et al. Ultra-processed food intake in association with BMI change and risk of overweight and obesity: A prospective analysis of the French NutriNet-Sante cohort. PLoS medicine. 2020;17(8):e1003256.
6. Blanco-Rojo R, Sandoval-Insausti H, Lopez-Garcia E, Graciani A, Ordovas JM, Banegas JR, et al. Consumption of Ultra-Processed Foods and Mortality: A National Prospective Cohort in Spain. Mayo Clinic proceedings. 2019;94(11):2178-88.

7. Bonaccio M, Di Castelnuovo A, Costanzo S, De Curtis A, Persichillo M, Sofi F, et al. Ultra-processed food consumption is associated with increased risk of all-cause and cardiovascular mortality in the Moli-sani Study. American Journal of Clinical Nutrition. 2021;113(2):446-55.
8. Bonaccio M, Di Castelnuovo A, Ruggiero E, Costanzo S, Grosso G, De Curtis A, et al. Joint association of food nutritional profile by Nutri-Score front-of-pack label and ultra-processed food intake with mortality: Moli-sani prospective cohort study. Bmj. 2022;378:e070688.
9. Bonaccio M, Costanzo S, Castelnuovo AD, Persichillo M, Magnacca S, Curtis AD, et al. Ultra-processed Food Intake and All-cause and Cause-specific Mortality in Subjects with Type 2 Diabetes: Longitudinal Findings from the Moli-sani Study. Circulation. 2022;145.
10. Bonaccio M, Costanzo S, Di Castelnuovo A, Persichillo M, Magnacca S, De Curtis A, et al. Ultra-processed food intake and all-cause and cause-specific mortality in individuals with cardiovascular disease: the Moli-sani Study. European Heart Journal. 2022;43(3):213-24.
11. Bonaccio M, Di Castelnuovo A, Costanzo S, Ruggiero E, Esposito S, Persichillo M, et al. Ultraprocessed food consumption is associated with all-cause and cardiovascular mortality in participants with type 2 diabetes independent of diet quality: a prospective observational cohort study. Am J Clin Nutr. 2023;118(3):627-36.
12. Canella DS, Levy RB, Martins APB, Claro RM, Moubarac J-C, Baraldi LG, et al. Ultra-processed food products and obesity in Brazilian households (2008-2009). PLoS ONE. 2014;9(3):e92752.
13. Canhada SL, Luft VC, Giatti L, Duncan BB, Chor D, Fonseca MdJMd, et al. Ultra-processed foods, incident overweight and obesity, and longitudinal changes in weight and waist circumference: the Brazilian Longitudinal Study of Adult Health (ELSA-Brasil). Public Health Nutrition. 2020;23(4):1076-86.
14. Canhada SL, Vigo A, Luft VC, Levy RB, Alvim Matos SM, Del Carmen Molina M, et al. Ultra-Processed Food Consumption and Increased Risk of Metabolic Syndrome in Adults: The ELSA-Brasil. Diabetes Care. 2023;46(2):369-76.
15. R Cardoso B, Machado P, Steele EM. Association between ultra-processed food consumption and cognitive performance in US older adults: a cross-sectional analysis of the NHANES 2011–2014. Eur J Nutr. 2022;61(8):3975-85.
16. Chang K, Gunter MJ, Rauber F, Levy RB, Huybrechts I, Kliemann N, et al. Ultra-processed food consumption, cancer risk and cancer mortality: a large-scale prospective analysis within the UK Biobank. EClinicalMedicine. 2023;56:101840.
17. Chen X, Chu J, Hu W, Sun N, He Q, Liu S, et al. Associations of ultra-processed food consumption with cardiovascular disease and all-cause mortality: UK Biobank. Eur J Public Health. 2022;32(5):779-85.
18. Chen J, Wellens J, Kalla R, Fu T, Deng M, Zhang H, et al. Intake of Ultra-processed Foods Is Associated with an Increased Risk of Crohn's Disease: A Cross-sectional and Prospective Analysis of 187 154 Participants in the UK Biobank. J Crohns Colitis. 2023;17(4):535-52.
19. Cordova R, Kliemann N, Huybrechts I, Rauber F, Vamos EP, Levy RB, et al. Consumption of ultra-processed foods associated with weight gain and obesity in adults: A multi-national cohort study. Clinical nutrition (Edinburgh, Scotland). 2021;40(9):5079-88.
20. Cross AJ, Gunter MJ. Ultra-processed foods and colorectal neoplasia: is there a link? Journal of the National Cancer Institute. 2023;115(2):117-9.
21. da Silva A, Brum Felício M, Caldas APS, Hermsdorff HH, Torreglosa CR, Bersch-Ferreira Â, et al. Ultra-processed foods consumption is associated with cardiovascular disease and cardiometabolic risk factors in Brazilians with established cardiovascular events. International journal of food sciences and nutrition. 2021;72(8):1128-37.
22. Silva CLd, Sousa AG, Borges LPSL, Costa THMd. Usual consumption of ultra-processed foods and its association with sex, age, physical activity, and body mass index in adults living in Brasilia City, Brazil. Revista brasileira de epidemiologia = Brazilian journal of epidemiology. 2021;24:e210033.

23. da Silva EC, Carneiro JR, de Almeida Fonseca Viola PC, Confortin SC, da Silva AAM. Association of Food Intake with Sleep Durations in Adolescents from a Capital City in Northeastern Brazil. Nutrients. 2022;14(23).
24. Detopoulou P, Dedes V, Pylarinou I, Syka D, Tzirogiannis K, Panoutsopoulos GI. Dietary acid load is associated with waist circumference in university students with low adherence to the Mediterranean diet: The potential role of ultra-processed foods. Clin Nutr ESPEN. 2023;56:43-51.
25. Detopoulou P, Dedes V, Syka D, Tzirogiannis K, Panoutsopoulos GI. Relation of Minimally Processed Foods and Ultra-Processed Foods with the Mediterranean Diet Score, Time-Related Meal Patterns and Waist Circumference: Results from a Cross-Sectional Study in University Students. Int J Environ Res Public Health. 2023;20(4):04.
26. Dehghan M, Mente A, Rangarajan S, Mohan V, Swaminathan S, Avezum A, et al. Ultra-processed foods and mortality: analysis from the Prospective Urban and Rural Epidemiology study. Am J Clin Nutr. 2023;117(1):55-63.
27. Donat-Vargas C, Sandoval-Insausti H, Rey-Garcia J, Moreno-Franco B, Akesson A, Banegas JR, et al. High Consumption of Ultra-Processed Food is Associated with Incident Dyslipidemia: A Prospective Study of Older Adults. J Nutr. 2021;151(8):2390-8.
28. Du S, Kim H, Rebholz CM. Higher Ultra-Processed Food Consumption Is Associated with Increased Risk of Incident Coronary Artery Disease in the Atherosclerosis Risk in Communities Study. J Nutr. 2021;151(12):3746-54.
29. Duan M-J, Vinke PC, Navis G, Corpeleijn E, Dekker LH. Ultra-processed food and incident type 2 diabetes: studying the underlying consumption patterns to unravel the health effects of this heterogeneous food category in the prospective Lifelines cohort. BMC Medicine. 2022;20(1):1-11.
30. El Kinany K, Huybrechts I, Hatime Z, El Asri A, Boudouaya HA, Deoula MMS, et al. Food processing groups and colorectal cancer risk in Morocco: evidence from a nationally representative case-control study. Eur J Nutr. 2022;61(5):2507-15.
31. Esposito S, Bonaccio M, Ruggiero E, Costanzo S, Di Castelnuovo A, Gialluisi A, et al. Food processing and risk of central nervous system tumours: A preliminary case-control analysis from the MEditerranean DIet in relation to CancEr of brAin (MEDICEA) study. Clin Nutr. 2023;42(2):93-101.
32. Fiolet T, Srour B, Sellem L, Kesse-Guyot E, All, egrave, et al. Consumption of ultra-processed foods and cancer risk: results from NutriNet-Santé prospective cohort. BMJ (Clinical research ed). 2018;360:k322.
33. Fitzpatrick JA, Halmos EP, Gibson PR, Machado PP. Ultra-processed Foods and Risk of Crohn's Disease: How Much is Too Much? Clin Gastroenterol Hepatol. 2023;21(10):2478-80.
34. Godos J, Bonaccio M, Al-Qahtani WH, Marx W, Lane MM, Leggio GM, et al. Ultra-Processed Food Consumption and Depressive Symptoms in a Mediterranean Cohort. Nutrients. 2023;15(3):18.
35. Gomez-Donoso C, Sanchez-Villegas A, Martinez-Gonzalez MA, Gea A, Mendonca RD, Lahortiga-Ramos F, et al. Ultra-processed food consumption and the incidence of depression in a Mediterranean cohort: the SUN Project. Eur J Nutr. 2020;59(3):1093-103.
36. Gourd E. Ultra-processed foods might increase cancer risk. Lancet Oncol. 2018;19(4):e186.
37. Haghighatdoost F, Atefi M, Mohammadifard N, Daryabeygi-Khotbehsara R, Khosravi A, Mansourian M. The relationship between ultraprocessed food consumption and obesity indicators in Iranian adults. Nutr Metab Cardiovasc Dis. 2022;32(9):2074-85.
38. Hall KD, Ayuketah A, Brychta R, Cai H, Cassimatis T, Chen KY, et al. Ultra-Processed Diets Cause Excess Calorie Intake and Weight Gain: An Inpatient Randomized Controlled Trial of Ad Libitum Food Intake. Cell Metab. 2019;30(1):67-77.e3.
39. Hang D, Wang L, Fang Z, Du M, Wang K, He X, et al. Ultra-processed food consumption and risk of colorectal cancer precursors: results from 3 prospective cohorts. Journal of the National Cancer Institute. 2023;115(2):155-64.
40. Hosseininasab D, Shiraseb F, Noori S, Jamili S, Mazaheri-Eftekhar F, Dehghan M, et al. The relationship between ultra-processed food intake and cardiometabolic risk factors in overweight and obese women: A cross-sectional study. Front. 2022;9:945591.

41. Ivancovsky-Wajcman D, Fliss-Isakov N, Webb M, Bentov I, Shibolet O, Kariv R, et al. Ultra-processed food is associated with features of metabolic syndrome and non-alcoholic fatty liver disease. Liver international : official journal of the International Association for the Study of the Liver. 2021;41(11):2635-45.
42. Jacobs I, Taljaard-Krugell C, Wicks M, Cubasch H, Joffe M, Laubscher R, et al. Degree of food processing and breast cancer risk in black urban women from Soweto, South African: the South African Breast Cancer study. Br J Nutr. 2022;128(11):2278-89.
43. Jafari F, Yarmand S, Nouri M, Nejad ET, Ramezani A, Sohrabi Z, et al. Ultra-Processed Food Intake and Risk of Colorectal Cancer: A Matched Case-Control Study. Nutr Cancer. 2023;75(2):532-41.
44. Juul F, Martinez-Steele E, Parekh N, Monteiro CA, Chang VW. Ultra-processed food consumption and excess weight among US adults. Br J Nutr. 2018;120(1):90-100.
45. Juul F, Vaidean G, Lin Y, Deierlein AL, Parekh N. Ultra-Processed Foods and Incident Cardiovascular Disease in the Framingham Offspring Study. Journal of the American College of Cardiology. 2021;77(12):1520-31.
46. Kim H, Hu EA, Rebholz CM. Ultra-processed food intake and mortality in the USA: results from the Third National Health and Nutrition Examination Survey (NHANES III, 1988-1994). Public Health Nutrition. 2019;22(10):1777-85.
47. Kim C, Na W, Choi S, Hwang SH, Sohn C. Relationship between metabolic syndrome and intake of ultra-processed foods in Korean adults: based on 6th and 7th Korea National Health and Nutrition Examination Survey (2013-2018). Nutr. 2023;17(4):735-46.
48. Kityo A, Lee SA. The intake of ultra-processed foods, all-cause, cancer and cardiovascular mortality in the Korean Genome and Epidemiology Study-Health Examinees (KoGES-HEXA) cohort. PLoS ONE. 2023;18(5):e0285314.
49. Kliemann N, Rauber F, Bertazzi Levy R, Viallon V, Vamos EP, Cordova R, et al. Food processing and cancer risk in Europe: results from the prospective EPIC cohort study. Lancet Planet Health. 2023;7(3):e219-e32.
50. Konieczna J, Morey M, Abete I, Bes-Rastrollo M, Ruiz-Canela M, Vioque J, et al. Contribution of ultra-processed foods in visceral fat deposition and other adiposity indicators: Prospective analysis nested in the PREDIMED-Plus trial. Clinical nutrition (Edinburgh, Scotland). 2021;40(6):4290-300.
51. Lee HY. Ultra-Processed Foods as a Less-Known Risk Factor in Cardiovascular Diseases. Korean circ. 2022;52(1):71-3.
52. Levy RB, Rauber F, Chang K, Louzada M, Monteiro CA, Millett C, et al. Ultra-processed food consumption and type 2 diabetes incidence: A prospective cohort study. Clin Nutr. 2021;40(5):3608-14.
53. Li M, Shi Z. Ultra-Processed Food Consumption Associated with Overweight/Obesity among Chinese Adults-Results from China Health and Nutrition Survey 1997-2011. Nutrients. 2021;13(8).
54. Li H, Li S, Yang H, Zhang Y, Ma Y, Hou Y, et al. Association of Ultra-Processed Food Intake with Cardiovascular and Respiratory Disease Multimorbidity: A Prospective Cohort Study. Mol Nutr Food Res. 2023;67(11):e2200628.
55. Liu Z, Huang H, Zeng Y, Chen Y, Xu C. Association between ultra-processed foods consumption and risk of non-alcoholic fatty liver disease: a population-based analysis of NHANES 2011-2018. Br J Nutr. 2023;130(6):996-1004.
56. Liu J, Steele EM, Li Y, Yi SS, Monteiro CA, Mozaffarian D. Consumption of Ultraprocessed Foods and Body Fat Distribution Among U.S. Adults. Am J Prev Med. 2023;65(3):427-38.
57. Llavero-Valero M, Escalada-San Martin J, Martinez-Gonzalez MA, Basterra-Gortari FJ, de la Fuente-Arrillaga C, Bes-Rastrollo M. Ultra-processed foods and type-2 diabetes risk in the SUN project: A prospective cohort study. Clin Nutr. 2021;40(5):2817-24.
58. Lo C-H, Khandpur N, Rossato SL, Lochhead P, Lopes EW, Burke KE, et al. Ultra-processed Foods and Risk of Crohn's Disease and Ulcerative Colitis: A Prospective Cohort Study. Clinical gastroenterology and hepatology : the official clinical practice journal of the American Gastroenterological Association. 2022;20(6):e1323-e37.

59. Louzada ML, Steele EM, Rezende LFM, Levy RB, Monteiro CA. Corrigendum: Changes in Obesity Prevalence Attributable to Ultra-Processed Food Consumption in Brazil Between 2002 and 2009. International Journal of Public Health. 2022;67:1605178.
60. Machado PP, Steele EM, Levy RB, da Costa Louzada ML, Rangan A, Woods J, et al. Ultra-processed food consumption and obesity in the Australian adult population. Nutrition & diabetes. 2020;10(1):39.
61. Magalhaes EIdS, de Oliveira BR, Rudakoff LCS, de Carvalho VA, Viola PCdAF, Arruda SPM, et al. Sex-Dependent Effects of the Intake of NOVA Classified Ultra-Processed Foods on Syndrome Metabolic Components in Brazilian Adults. Nutrients. 2022;14(15).
62. Martinez-Perez C, San-Cristobal R, Guallar-Castillon P, Martinez-Gonzalez MA, Salas-Salvado J, Corella D, et al. Use of Different Food Classification Systems to Assess the Association between Ultra-Processed Food Consumption and Cardiometabolic Health in an Elderly Population with Metabolic Syndrome (PREDIMED-Plus Cohort). Nutrients. 2021;13(7):20.
63. Martinez Steele E, Juul F, Neri D, Rauber F, Monteiro CA. Dietary share of ultra-processed foods and metabolic syndrome in the US adult population. Prev Med. 2019;125:40-8.
64. Martinez Steele E, Marron Ponce JA, Cediel G, Louzada MLC, Khandpur N, Machado P, et al. Potential reductions in ultra-processed food consumption substantially improve population cardiometabolic-related dietary nutrient profiles in eight countries. Nutr Metab Cardiovasc Dis. 2022;32(12):2739-50.
65. Mendonca RD, Pimenta AM, Gea A, de la Fuente-Arrillaga C, Martinez-Gonzalez MA, Lopes AC, et al. Ultraprocessed food consumption and risk of overweight and obesity: the University of Navarra Follow-Up (SUN) cohort study. American Journal of Clinical Nutrition. 2016;104(5):1433-40.
66. Mendonca RD, Lopes AC, Pimenta AM, Gea A, Martinez-Gonzalez MA, Bes-Rastrollo M. Ultra-Processed Food Consumption and the Incidence of Hypertension in a Mediterranean Cohort: The Seguimiento Universidad de Navarra Project. Am J Hypertens. 2017;30(4):358-66.
67. Monge A, Lajous M. Ultra-processed foods and cancer. BMJ (Clinical research ed). 2018;360:k599.
68. Monge A, Silva Canella D, Lopez-Olmedo N, Lajous M, Cortes-Valencia A, Stern D. Ultraprocessed beverages and processed meats increase the incidence of hypertension in Mexican women. Br J Nutr. 2021;126(4):600-11.
69. Montero-Salazar H, Donat-Vargas C, Moreno-Franco B, Sandoval-Insausti H, Civeira F, Laclaustra M, et al. High consumption of ultra-processed food may double the risk of subclinical coronary atherosclerosis: the Aragon Workers' Health Study (AWHS). BMC Medicine. 2020;18(1):1-11.
70. Vasconcelos Leitao Moreira P, da Costa Pereira de Arruda Neta A, Leite de Lima Ferreira FE, de Araujo JM, da Costa Louzada ML, Lira Formiga Cavalcanti de Lima R, et al. Projected impact of change in the percentage of energy from each NOVA group intake on cardiovascular disease mortality in Brazil: a modelling study. BMJ open. 2022;12(4):e057953.
71. Mullen A. Ultraprocessed food and cardiometabolic disease. Nat Food. 2021;2(8):554.
72. Nardocci M, Polsky JY, Moubarac J-C. Consumption of ultra-processed foods is associated with obesity, diabetes and hypertension in Canadian adults. Canadian journal of public health = Revue canadienne de sante publique. 2021;112(3):421-9.
73. Narula N, Wong ECL, Dehghan M, Mente A, Rangarajan S, Lanas F, et al. Association of ultra-processed food intake with risk of inflammatory bowel disease: prospective cohort study. BMJ (Clinical research ed). 2021;374:n1554.
74. Nilson EAF, Ferrari G, Louzada M, Levy RB, Monteiro CA, Rezende LFM. The estimated burden of ultra-processed foods on cardiovascular disease outcomes in Brazil: A modeling study. Front. 2022;9:1043620.
75. Osté MCJ, Duan M-J, Gomes-Neto AW, Vinke PC, Carrero J-J, Avesani C, et al. Ultra-processed foods and risk of all-cause mortality in renal transplant recipients. American Journal of Clinical Nutrition. 2022;115(6):1646-57.

76. Pang T, Gray HL, Alman AC, Buro AW, Basu A, Lu S, et al. Ultra-processed food consumption and obesity indicators in individuals with and without type 1 diabetes mellitus: a longitudinal analysis of the prospective Coronary Artery Calcification in Type 1 Diabetes (CACTI) cohort study. Public Health Nutrition. 2023;26(8):1626-33.
77. Peres SV, Silva DRM, Coimbra FJF, Fagundes MA, Auzier JJN, Pelosof AG, et al. Consumption of processed and ultra-processed foods by patients with stomach adenocarcinoma: a multicentric case-control study in the Amazon and southeast regions of Brazil. Cancer causes & control : CCC. 2022;33(6):889-98.
78. Pestoni G, Habib L, Reber E, Rohrmann S, Staub K, Stanga Z, et al. Ultraprocessed Food Consumption is Strongly and Dose-Dependently Associated with Excess Body Weight in Swiss Women. Obesity (Silver Spring). 2021;29(3):601-9.
79. Rauber F, Chang K, Vamos EP, da Costa Louzada ML, Monteiro CA, Millett C, et al. Ultra-processed food consumption and risk of obesity: a prospective cohort study of UK Biobank. Eur J Nutr. 2021;60(4):2169-80.
80. Rezende-Alves K, Hermsdorff HHM, Miranda AEdS, Lopes ACS, Bressan J, Pimenta AM. Food processing and risk of hypertension: Cohort of Universities of Minas Gerais, Brazil (CUME Project). Public health nutrition. 2021;24(13):4071-9.
81. Rico-Campà A, Martínez-González MA, Alvarez-Alvarez I, Mendonça RD, de la Fuente-Arrillaga C, Gómez-Donoso C, et al. Association between consumption of ultra-processed foods and all cause mortality: SUN prospective cohort study. Bmj. 2019;365:l1949.
82. Romaguera D, Fernandez-Barres S, Gracia-Lavedan E, Vendrell E, Azpiri M, Ruiz-Moreno E, et al. Consumption of ultra-processed foods and drinks and colorectal, breast, and prostate cancer. Clinical nutrition (Edinburgh, Scotland). 2021;40(4):1537-45.
83. Romieu I, Khandpur N, Katsikari A, Biessy C, Torres-Mejia G, Angeles-Llerenas A, et al. Consumption of industrial processed foods and risk of premenopausal breast cancer among Latin American women: the PRECAMA study. BMJ nutr. 2022;5(1):1-9.
84. Sandoval-Insausti H, Jiménez-Onsurbe M, Donat-Vargas C, Rey-García J, Banegas JR, Rodríguez-Artalejo F, et al. Ultra-Processed Food Consumption Is Associated with Abdominal Obesity: A Prospective Cohort Study in Older Adults. Nutrients. 2020;12(8):2368.
85. Santos AC, Passos AFF, de Souza LB, Coelho ASG, Cominetti C. Consumption of ultra- and non-ultra-processed foods of individuals with normal-weight obesity. Journal of Nutritional Science. 2023;12:e71.
86. Scaranni P, Cardoso LO, Chor D, Melo ECP, Matos SMA, Giatti L, et al. Ultra-processed foods, changes in blood pressure and incidence of hypertension: the Brazilian Longitudinal Study of Adult Health (ELSA-Brasil). Public Health Nutr. 2021;24(11):3352-60.
87. Schnabel L, Buscail C, Sabate J-M, Bouchoucha M, Kesse-Guyot E, Alles B, et al. Association Between Ultra-Processed Food Consumption and Functional Gastrointestinal Disorders: Results From the French NutriNet-Sante Cohort. The American journal of gastroenterology. 2018;113(8):1217-28.
88. Schnabel L, Kesse-Guyot E, Alles B, Touvier M, Srour B, Hercberg S, et al. Association Between Ultraprocessed Food Consumption and Risk of Mortality Among Middle-aged Adults in France. JAMA Internal Medicine. 2019;179(4):490-8.
89. Shim SY, Kim HC, Shim JS. Consumption of Ultra-Processed Food and Blood Pressure in Korean Adults. Korean circ. 2022;52(1):60-70.
90. Shim JS, Ha KH, Kim DJ, Kim HC. Ultra-Processed Food Consumption and Obesity in Korean Adults. Diabetes & Metabolism Journal. 2023;47(4):547-58.
91. Silva FM, Giatti L, de Figueiredo RC, Molina MDCB, de Oliveira Cardoso L, Duncan BB, et al. Consumption of ultra-processed food and obesity: cross sectional results from the Brazilian Longitudinal Study of Adult Health (ELSA-Brasil) cohort (2008-2010). Public health nutrition. 2018;21(12):2271-9.
92. Silva LV, Abdalla PP, Bohn L, Araujo RG, Batalhao DF, Venturini ACR, et al. Association of minimally processed and ultra-processed food daily consumption with obesity in overweight adults: a cross-sectional study. Nutr Hosp. 2023;40(3):534-42.

93. Smaira FI, Mazzolani BC, Pecanha T, Dos Santos KM, Rezende DAN, Araujo ME, et al. Ultra-processed food consumption associates with higher cardiovascular risk in rheumatoid arthritis. Clin Rheumatol. 2020;39(5):1423-8.
94. Smiljanec K, Mbakwe AU, Ramos-Gonzalez M, Mesbah C, Lennon SL. Associations of Ultra-Processed and Unprocessed/Minimally Processed Food Consumption with Peripheral and Central Hemodynamics and Arterial Stiffness in Young Healthy Adults. Nutrients. 2020;12(11):3229.
95. Srour B, Fezeu LK, Kesse-Guyot E, Allès B, Méjean C, Andrianasolo RM, et al. Ultra-processed food intake and risk of cardiovascular disease: prospective cohort study (NutriNet-Santé). Bmj. 2019;365:l1451.
96. Srour B, Fezeu LK, Kesse-Guyot E, Alles B, Debras C, Druesne-Pecollo N, et al. Ultraprocessed Food Consumption and Risk of Type 2 Diabetes Among Participants of the NutriNet-Sante Prospective Cohort. JAMA Internal Medicine. 2020;180(2):283-91.
97. Sun M, He Q, Li G, Zhao H, Wang Y, Ma Z, et al. Association of ultra-processed food consumption with incident depression and anxiety: a population-based cohort study. Food Funct. 2023;14(16):7631-41.
98. Trudeau K, Rousseau M-C, Parent M-E. Extent of Food Processing and Risk of Prostate Cancer: The PROtEuS Study in Montreal, Canada. Nutrients. 2020;12(3).
99. Vandevijvere S, Jaacks LM, Monteiro CA, Moubarac JC, Girling-Butcher M, Lee AC, et al. Global trends in ultraprocessed food and drink product sales and their association with adult body mass index trajectories. Obes Rev. 2019;20 Suppl 2:10-9.
100. Vasseur P, Dugelay E, Benamouzig R, Savoye G, Lan A, Srour B, et al. Dietary Patterns, Ultra-processed Food, and the Risk of Inflammatory Bowel Diseases in the NutriNet-Sante Cohort. Inflammatory bowel diseases. 2021;27(1):65-73.
101. Vellinga RE, van den Boomgaard I, J MAB, van der Schouw YT, Harbers MC, Verschuren WMM, et al. Different Levels of Ultraprocessed Food and Beverage Consumption and Associations with Environmental Sustainability and All-cause Mortality in EPIC-NL. Am J Clin Nutr. 2023;118(1):103-13.
102. Yang Q, Zhang Z, Steele EM, Moore LV, Jackson SL. Ultra-Processed Foods and Excess Heart Age Among U.S. Adults. Am J Prev Med. 2020;59(5):e197-e206.
103. Zhang T, Gan S, Ye M, Meng G, Zhang Q, Liu L, et al. Association between consumption of ultra-processed foods and hyperuricemia: TCLSIH prospective cohort study. Nutrition, Metabolism and Cardiovascular Diseases. 2021;31(7):1993-2003.
104. Zhang S, Gan S, Zhang Q, Liu L, Meng G, Yao Z, et al. Ultra-processed food consumption and the risk of non-alcoholic fatty liver disease in the Tianjin Chronic Low-grade Systemic Inflammation and Health Cohort Study. Int J Epidemiol. 2022;51(1):237-49.
105. Zhong GC, Zhu Q, Cai D, Hu JJ, Dai X, Gong JP, et al. Ultra-processed food consumption and the risk of pancreatic cancer in the Prostate, Lung, Colorectal and Ovarian Cancer Screening Trial. Int J Cancer. 2023;152(5):835-44.
106. Kelly SE, Greene-Finestone LS, Yetley EA, Benkhedda K, Brooks SPJ, Wells GA, et al. NUQUEST-NUtrition QUality Evaluation Strengthening Tools: development of tools for the evaluation of risk of bias in nutrition studies. Am J Clin Nutr. 2022;115(1):256-71.
